# Jack of all trades, master of one: domain-specific and domain-general contributions to perceptual expertise in visual comparison

**DOI:** 10.1186/s41235-024-00596-0

**Published:** 2024-10-29

**Authors:** Bethany Growns, James D. Dunn, Rebecca K. Helm, Alice Towler, Erwin J. A. T. Mattijssen, Kristy A. Martire

**Affiliations:** 1 School of Psychology, Speech and Hearing, University of Canterbury, Christchurch, New Zealand; 2 Law School, University of Exeter, Exeter, UK; 3 School of Psychology, University of New South Wales, Sydney, Australia; 4 School of Psychology, University of Queensland, Brisbane, Australia; 5 The Netherlands Forensic Institute, The Hague, The Netherlands

**Keywords:** Perceptual expertise, Individual differences, Expertise, Domain-general, Forensic science, Visual comparison, Pattern-matching

## Abstract

Perceptual expertise is typically domain-specific and rarely generalises beyond an expert’s domain of experience. Forensic feature-comparison examiners outperform the norm in domain-specific visual comparison, but emerging research suggests that they show advantages on other similar tasks outside their domain of expertise. For example, fingerprint examiners not only outperform novices in fingerprint comparison, but also in face comparison. Yet, the extent to which their skills generalise is poorly understood. In this study, we investigated the generalisability of perceptual expertise amongst forensic examiners by comparing their performance to novices and other examiners within and outside their area of expertise. We recruited 85 experts from three forensic disciplines (face, fingerprint, and firearms) and asked them to complete four different visual comparison tasks: faces, fingerprints, firearms, and novel-objects. Examiners displayed domain-specific expertise: they outperformed novices and other examiners within their domain of visual comparison expertise. Yet, some of their skill also generalised: examiners also outperformed novices outside their area of expertise. However, while individual differences in examiners’ performance within their domain of experience were associated with their performance in a novel comparison task, they were not related to their performance on tasks outside their expert domain. These results provide key insight into the domain-specific and domain-general contributions of forensic examiners’ perceptual expertise. Forensic expertise lends some generalisable skill to other visual comparison tasks, but best performance is still seen within examiners’ domain of expertise.

## Significance statement

The ability to spot differences or similarities between patterns—like comparing fingerprints or recognising faces—varies widely. Forensic science examiners in select disciplines excel in this in tasks within their domain of expertise (e.g., a fingerprint examiner comparing fingerprints). However, how they fare outside their domain of expertise is less well understood (e.g., a fingerprint examiner comparing faces). In this study, we recruited face, fingerprint, and firearms examiners to explore if their skill generalises beyond their domain of expertise. We found a hierarchy of expert performance: examiners outperformed other examiners and novices within their domain of expertise but also outperformed novices outside their domain. Examiners’ skill does generalise, but only to a certain extent. As accuracy is maximised in an examiner’s domain of expertise, our results do not suggest that professional performance would be improved by examiners practicing outside their trained discipline, but rather that examiners possess or acquire an ability that partially generalises across domains through training or experience. This implies that there may be common mechanisms underpinning the generalisation of visual comparison skills across domains. Future research could uncover these mechanisms to use in training programmes and develop evidence-based programmes to fast-track the performance of new trainees. Our results also suggest that there is individual variation in skill amongst both professionals and novices. Forensic science organisations could also improve professional performance by recruiting people with a natural aptitude for visual comparison from the general population.

## Introduction

Expertise is typically characterised as narrow and domain-specific (Bedard & Chi, 1992; Ericsson et al., 1993, 2018). It is thought that expert skill is developed via experience and deliberate practice within an expert’s primary domain of expertise (Ericsson, 2007; Ericsson et al., 1993; Keith & Ericsson, 2007). Popular culture estimates suggest that it takes 10,000 h to become an expert (Gladwell, 2008). It has thus long been thought that ‘experts excel mainly in their own domain’ (Chi et al., 1988, p. xvii). Many studies confirm that domain-specific expertise rarely generalises beyond an expert’s domain of experience. For example, orthodontists can judge face symmetry better than novices, but not non-face stimuli (Jackson et al., 2013), super-memorizers, those with superior associative memory, do not have superior face recognition memory (Ramon et al., 2016), and modern car experts can discriminate modern cars better than novices, but not antique cars (Bukach et al., 2010).

Yet, many other factors are at play in how expertise develops, such as individual differences in talent, cognitive abilities, genetics, and personality (Hambrick et al., 2016). The acquisition of expertise interacts with individual differences and domain-general abilities in many different disciplines where some people acquire expertise faster in a given domain than others (Kaufman, 2007). This is seen in experts in domains like bird or mineral expertise (Martens et al., 2018) to chess expertise (Smith et al., 2021). If interaction between individual differences and the acquisition of expertise is also generalisable across domains, it could be used to predict subsequent expertise.

One area with important real-world consequences where domain-*general* individual differences and domain-*specific* expertise interact is forensic visual comparison (Growns et al., 2022a; Phillips et al., 2018). Visual comparison is a complex task where visual stimuli are compared to determine whether they are from the same or different sources. Forensic examiners like face, fingerprint, and firearms examiners complete visual comparison tasks professionally to link or exclude evidence from crime scenes (National Academy of Sciences, 2009; President’s Council of Advisors on Science and Technology, 2016). For example, face examiners compare images of faces to identify suspects of crime in CCTV or prevent passport fraud (White et al., 2020). Similarly, fingerprint examiners compare fingerprints found at crime scenes to judge whether they are from a specific suspect or a different person (Busey & Vanderkolk, 2005), and firearms examiners compare cartridge cases fired from guns to link a specific bullet to a specific gun (Mattijssen et al., 2020). Face, fingerprint, and firearms examiners possess domain-specific perceptual expertise as they outperform the norm in visual comparison within their area of experience (Busey & Vanderkolk, 2005; Gutierrez & Prokesch, 2024; Mattijssen et al., 2020; Tangen et al., 2011; White et al., 2015b).

Yet, there is also emerging evidence of domain-general ability amongst some forensic examiners. Fingerprint examiners outperform novices in face comparison, a task outside their domain of expertise (Phillips et al., 2018). Similarly, face examiners outperform novices in fingerprint comparison, also a task beyond their expertise (Towler et al., 2023). This suggests that the perceptual expertise of forensic examiners may lend generalise skill and enable above-average performance in domains outside of their expertise. It is possible that individual differences in visual comparison interact with the acquisition of expertise in ways that are not yet understood.

Individual differences in visual comparison are seen even amongst professional forensic examiners. For example, face examiners’ area under the curve (AUC) scores on a proficiency test designed to reflect professional casework ranged from 0.72 to 0.99 (Towler et al., 2023; see also Sexton et al., 2024). Similarly, fingerprint trainees’ performance on fingerprint comparison varies from 77% to 87% accuracy even after 12 months of training (Searston & Tangen, 2017b). Face examiners’ face comparison ability is also not correlated with their length of employment (White et al., 2015a), and variation in performance is also seen amongst firearms examiners (Gutierrez & Prokesch, 2024; Mattijssen et al., 2020). Further, individual differences in skill amongst forensic trainees before training are reliable predictors of future professional performance in fingerprint comparison (Searston & Tangen, 2017b). Even in the general population, there is variation in visual comparison ability amongst those without forensic science training or experience (Growns et al., 2022a). Novices’ visual comparison skill generalises across different complex visual stimuli: top-performing novices who excel at comparing one type of stimuli (e.g., fingerprints) also excel with other types of stimuli (e.g. faces or firearms; Growns et al., 2022a).

Pre-existing individual differences in visual comparison ability in the general population may interact with the development of expertise in forensic science in ways that remain unclear. Pre-existing variation in skill may be one reason that forensic examiners’ ability generalises beyond their domain of expertise. Yet, this is difficult to explore as the development of expertise can also *reduce* existing variance in ability. We thus aim to explore how the perceptual expertise of forensic examiners generalises across domains at both a group and individual level, across a range of domain-specific, domain-general, and entirely novel tasks.

In this paper, we investigate the relationship between domain-general cognitive abilities and expertise by exploring the domain-specific and domain-general contributions to forensic examiners’ perceptual expertise. Forensic expertise provides an opportunity to investigate the relationship between expertise and domain-general mechanisms as experts can make essentially the same judgement (i.e., same or different source) about different stimuli (i.e., those within and outside their domain of expertise). At a group level, we investigated whether forensic examiners from three different disciplines (faces, fingerprints, and firearms) outperform each other in their expert domain (i.e., domain-specific), non-expert-domain (i.e., domain-general), and an entirely novel visual comparison task. At an individual level, we examined whether individual differences in examiners’ expert-domain visual comparison performance were predicted by non-expert-domain ability, and by other personality (intrinsic motivation) and cognitive abilities (statistical learning).

## Method

We recruited face, fingerprint, and firearms examiners to complete four visual comparison tasks (face, fingerprints, firearms, and novel-objects) and two discriminant validity tasks (intrinsic motivation and statistical learning), with novices as a control comparison sample. The pre-registration, data, and analysis scripts can be found at https://osf.io/2ahsq/.

### Participants

Participants were 85 forensic examiners (13 face, 42 fingerprint, and 30 firearms)^1^ recruited via a snowball-sampling method with emails sent to forensic organisations and mailing lists. The sample size was determined by the number of forensic examiners recruited during our pre-registered data acquisition period. Forensic examiners first indicated whether or not they would describe themselves as a forensic scientist or practitioner, and then nominated the discipline that was their primary area of specialisation (i.e., face, fingerprint, firearms, or other discipline). They then provided information about their experience and employment within their primary area of specialisation (see Table 1).

**Table 1:** Sample size, demographic, and professional practice characteristics for each group

	*N*	*M* age (SD, range)	Gender	*M* years professional experience (SD, range)
Face examiners	13	36.3 (8.6, 24–54)	76.9% female, 23.1% male	6.5 (5.2, 1–14)
Fingerprint examiners	42	43.8 (10.4, 24–70)	61.9% female, 38.1% male	17.5 (11.1, 2–47)
Firearm examiners	30	45.4 (8.7, 28–65)	33.3% female, 66.7% male	13.3 (8.7, 1–34)
Novices	93	35.7 (10.2, 18–64)	50.5% female, 49.5% male	–

We then recruited 93 novices from Prolific Academic as a sample-size-matched comparison, including an additional 10% (*n* = 8) to account for attrition. Participants from Prolific were required to have normal or corrected-to-normal vision and an approval rate on Prolific of 95% or above. We elected to use a novice comparison sample for ease of recruitment, but it is important to note that previous research has shown that comparable professional samples without domain-specific training do not perform at the same level as experts. For example, lawyers do not outperform firearms examiners (Gutierrez & Prokesch, 2024), and facial reviewers who compare passport photos to detect passport fraud do not outperform face examiners who receive extensive training and mentorship in face comparison (White et al., 2015a, 2015b).

Novices were paid £6.50 for participation in the approximately 60-min study, examiners were not paid for their involvement. To motivate performance, all participants had the chance to win one of ten £500 Amazon vouchers that were awarded to the top two performers in each task, including statistical learning (except the intrinsic motivation inventory). Novices were not informed that examiners were also participating in this experiment to ensure their incentive to participate was not impaired. It is thus likely that compensation was comparably motivating for novices and examiners.

No participants were excluded based on our exclusion criteria of not passing at least three of four attention-check questions. Demographic and professional practice information for each group can be seen in Table 1.

## Materials

### Visual comparison tasks

#### Face comparison task

**Fig. 1:**
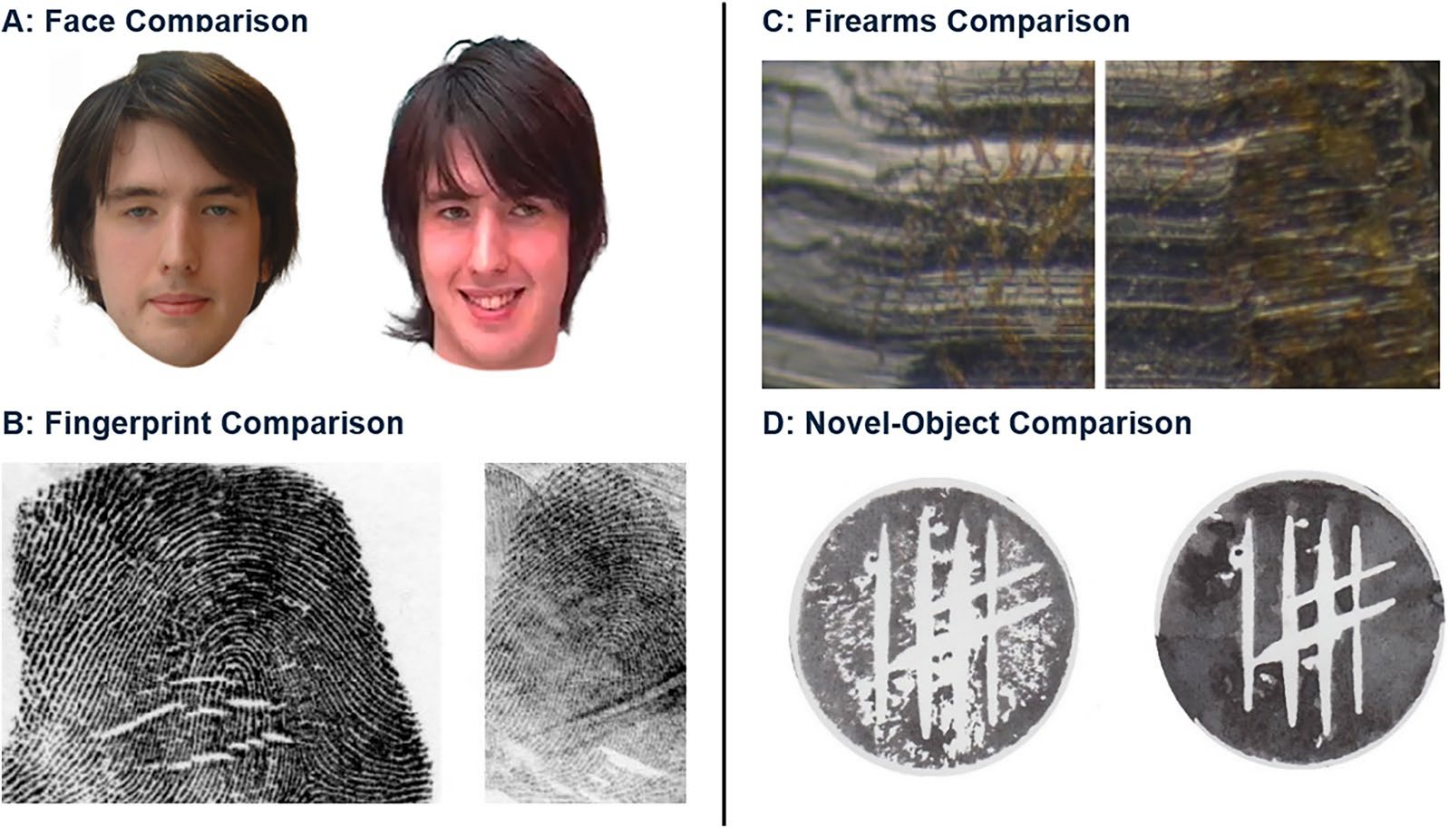
Example ‘match’ trials for each comparison task (Panel **A**: face comparison, Panel **B**: fingerprint comparison, Panel **C**: firearms comparison, and Panel **D**: novel-object comparison)

Participants completed 40 face comparison trials (20 match and 20 non-match)^2^ from the Glasgow Face-Matching Task 2—High-Version (GFMT2-High; White et al., 2021). Participants viewed two faces side-by-side and were asked ‘Are these images of the same person or two different people?’ on each trial. They responded by selecting one of two buttons (‘same’ or ‘different’) at the bottom of the screen (see Fig. 1). To best capture the skill of face comparison experts, we used the the GFMT2-high because it contains trials that are designed to discriminate between top-performers (see White et al., 2021). That is, trials in the GFMT2-High were selected based on the highest item-to-test correlations for individuals with above-median performance—or how well accuracy on each trial predicts a participant’s overall performance (Guilford, 1954; Wilmer et al., 2012).

#### Fingerprint comparison task

Participants completed 40 fingerprint comparison trials (20 match and 20 non-match) from the fingerprint comparison task in Growns et al. (2022a). Participants viewed two fingerprints side-by-side and were asked ‘Are these fingerprints from the same person or two different people?’ on each trial (see Fig. 1). They responded by selecting one of two buttons (‘same’ or ‘different’) at the bottom of the screen. To best measure the performance of fingerprint comparison experts, we selected trials using the same method as the GFMT2-High: 40 trials were chosen with the highest item-to-test correlations for individuals with above-median performance in previous research (i.e., Experiment 2 in Growns et al., 2022a).

#### Firearms comparison task

Participants completed 40 firearms comparison trials (20 match and 20 non-match) from the firearms comparison task in Growns et al. (2022a). Participants viewed two cartridge cases side-by-side and were asked ‘Are these cartridge cases from the same firearm or two different firearms?’ on each trial (see Fig. 1). They responded by selecting one of two buttons (‘same’ or ‘different’) at the bottom of the screen. To best measure the performance of firearms comparison experts, we selected trials using the same method as above of selecting 40 trials with the highest item-to-test correlations for above-median performance (i.e., in Experiment 2 in Growns et al., 2022a).

#### Novel-object comparison

Participants completed 40 novel-object comparison trials (20 match and 20 non-match) from the Novel-Object-Matching Test (Growns et al., 2023). Participants viewed two novel-objects side-by-side and were asked ‘Are these prints from the same stamping tool or two different stamping tools?’ on each trial (see Fig. 1). They responded by selecting one of two buttons (‘same’ or ‘different’) at the bottom of the screen. To best capture the performance of all experts in a non-expert-domain task, we selected trials using the same method as above of selecting 40 trials with the highest item-to-test correlations for above-median performance (i.e., in Experiment 2 in Growns et al., 2022a, 2022b, 2022c).

### Discriminant validity tasks

#### Intrinsic motivation inventory

Participants completed a measure of their intrinsic motivation and subjective experience during the experiment: the Intrinsic Motivation Inventory (McAuley et al., 1989). The Intrinsic Motivation Inventory is a validated measure of intrinsic motivation because it has acceptable reliability and stability (McAuley et al., 1989; Tsigilis & Theodosiou, 2003) and has been used across multiple domains—from education to mental health research (Choi et al., 2010; Leng et al., 2010; Monteiro et al., 2015). Participants completed three sub-scales of the inventory: the *effort, enjoyment*, and *perceived competence* sub-scales. They answered questions on a 7-point Likert scale from ‘Not at All True’ to ‘Very True’. They answered questions such as: ‘I put a lot of effort into this’ (effort sub-scale); ‘I enjoyed doing this activity very much’ (enjoyment sub-scale); and ‘I am satisfied with my performance in this task’ (perceived competence sub-scale). A full list of the questions can be found at https://selfdeterminationtheory.org/intrinsic-motivation-inventory/.

#### Statistical learning task

**Fig. 2:**
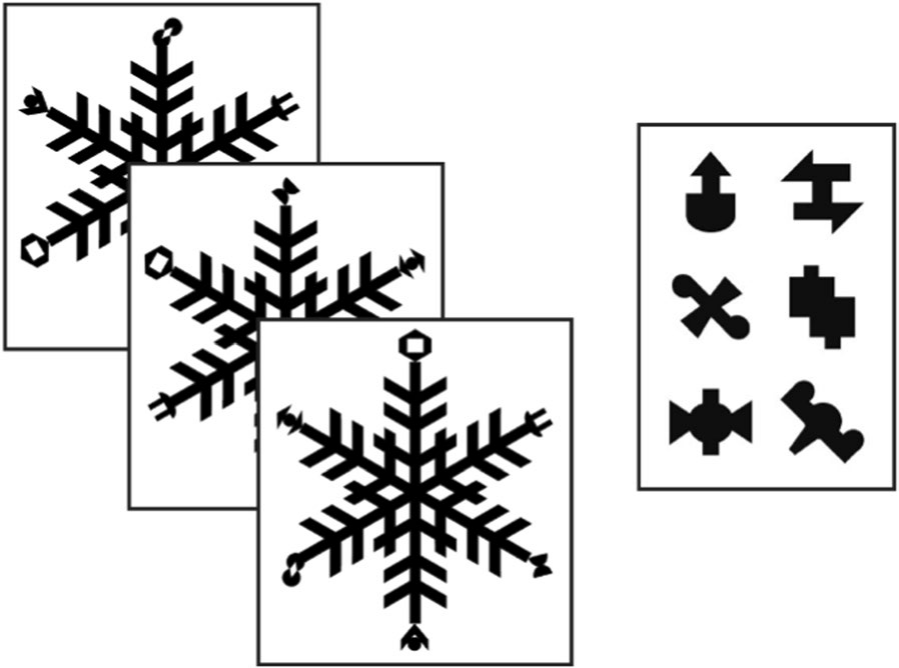
Visual example of one of the divergent validity statistical learning tasks. The image depicts three exemplars that participants viewed one after the other in the exposure phase on the left, with the six features that were manipulated to appear with different statistical frequencies

Participants completed a visual statistical learning task adapted from previous research (Growns & Martire, 2020a; Growns et al., 2020) where participants first completed an exposure phase and then a test phase. During the exposure phase, participants viewed 60 complex patterns (see Fig. 2) in a randomised order (each pattern displayed for 3-s with a 200-ms interval in-between) and were instructed to pay attention to them as they would be asked some questions about them afterwards. Each pattern contained different features (see Fig. 2) on the ends of the pattern ‘arms’ that occurred with different statistical frequencies across all patterns (e.g. feature ‘A’ appeared in 10% of patterns, while feature ‘B’ appeared in 20% of patterns). During the test phase, participants completed 45 trials where they were tested on how well they learned the frequencies, by being asked which of 2, 3, or 4 features were more familiar to them.

### Procedure

All participants completed the experiment via Qualtrics (2005). They first consented to participate in the study and then provided brief demographic and professional practice information (examiners only), received instructions, and then completed the five visual comparison tasks and statistical learning tasks in a random order, followed by the intrinsic motivation task. Finally, participants were debriefed.

### Dependent measures

Performance in each visual comparison task was measured using signal-detection measures of computed sensitivity and response bias (*d*′ and *C*; Phillips et al., 2001; Stanislaw & Todorov, 1999). To calculate sensitivity and bias in visual comparison, we coded hits as correct judgements on match trials and false alarms as incorrect judgements on non-match trials (see Phillips et al., 2001 for further discussion on the use of signal-detection measure in forensic science decision-making). Higher *d*′ values indicate higher sensitivity to the presence of a target stimulus independent of a tendency to respond ‘same’ or ‘different’ (response bias) and higher values are typically interpreted as higher ‘accuracy’ in a task. We also calculated participants’ criterion (*C*)—a measure of tendency to respond ‘same’ or ‘different’.

Intrinsic motivation scores were calculated by averaging participants’ Likert-scale responses on the *effort, enjoyment*, and *perceived competence* inventory sub-scales (including the reverse-scored items). Statistical learning scores were calculated by averaging the number of trials participants correctly chose the most frequent feature, where higher scores indicated better statistical learning.

### Analytical approach

We compared visual comparison sensitivity between expert-domain examiners, non-expert-domain examiners, and novices. We pre-registered our intention to recruit a specific sample size of examiners (*n* = 50 per group), but we did not reach this sample size during our pre-registered data collection period. We therefore adapted our analytical approach to maximise the number of participants in exploratory analyses by categorising each forensic examiner as either an expert domain examiner (e.g., a fingerprint examiner’s fingerprint comparison sensitivity) or a non-expert-domain examiner (e.g., a face or firearms examiner’s fingerprint sensitivity). For the exploratory group-level analyses reported in-text, we thus compared the sensitivity between expert-domain examiners, non-expert domain examiners, and novices. To account for unequal variances between groups, Welch corrections were applied using the *t.test* function in the base stats package in R to all follow-up comparisons (Delacre et al., 2017).

For the exploratory individual differences analyses reported in-text, we calculated Pearson’s correlations using the base stats package in R to investigate the relationship between examiners’ expert domain visual comparison sensitivity, their aggregate non-expert-domain sensitivity, and their novel visual comparison sensitivity (e.g., Novel-Object Matching sensitivity). We also calculated exploratory Bayes correlations using the *BayesFactor* package in R to examine the likelihood of the data under the null hypothesis (i.e., absence of correlations) compared to an alternative hypothesis (Morey et al., 2018). In our study, we combined multiple test scores into aggregate scores for our correlational analyses. To standardise these scores, we used Z-score transformations of sensitivity (*d*′) scores. Specifically, we calculated these Z-scores based on the mean (*M*) and standard deviation (SD) of novice examiners’ sensitivity. This approach ensured that the examiners’ sensitivity scores were standardised relative to the normative performance of novices. As we were primarily interested in how individual differences were affected by expertise, we computed these correlations for forensic examiners only.

## Results

### Descriptive statistics

The descriptive statistics for sensitivity of each group on each task can be seen in Table 2, and the psychometric properties of all tasks can be seen in Table 3 in the “Appendix”.

**Table 2:** Descriptive statistics for each task for all groups (*M*, SD in parentheses, *p* values for above chance *t*-tests)

	Novices		Face examiners		Fingerprint examiners		Firearms examiners	
	*M* (SD)	*p*	*M* (SD)	*p*	*M* (SD)	*p*	*M* (SD)	*p*
Face comparison	1.65 (.67)	< .001	2.19 (.37)	< .001	1.95 (.60)	< .001	1.86 (.52)	< .001
Fingerprint comparison	.38 (.62)	< .001	2.20 (.86)	< .001	2.91 (.61)	< .001	1.34 (.65)	< .001
Firearms comparison	2.31 (.94)	< .001	3.23 (.39)	< .001	3.14 (.57)	< .001	3.39 (.48)	< .001
Novel-object comparison	1.41 (.64)	< .001	2.47 (.68)	< .001	2.29 (.69)	< .001	2.20 (.65)	< .001
Intrinsic motivation	5.15 (.89)	–	5.09 (.87)	–	5.02 (.90)	–	4.37 (.73)	–
Statistical learning	0.48 (.19)	< .001	0.55 (.25)	< .001	0.52 (.22)	< .001	0.53 (.22)	< .001

Performance for face, fingerprint, firearms, and novel-objects are shown in *d*′, and average statistical learning and intrinsic motivation scores

### Exploratory group analyses

#### Visual comparison tasks

Analyses were conducted using the base stats package in R, and effect sizes (i.e., Cohen’s d) were calculated using the *lsr* package (Navarro, 2013).

**Fig. 3:**
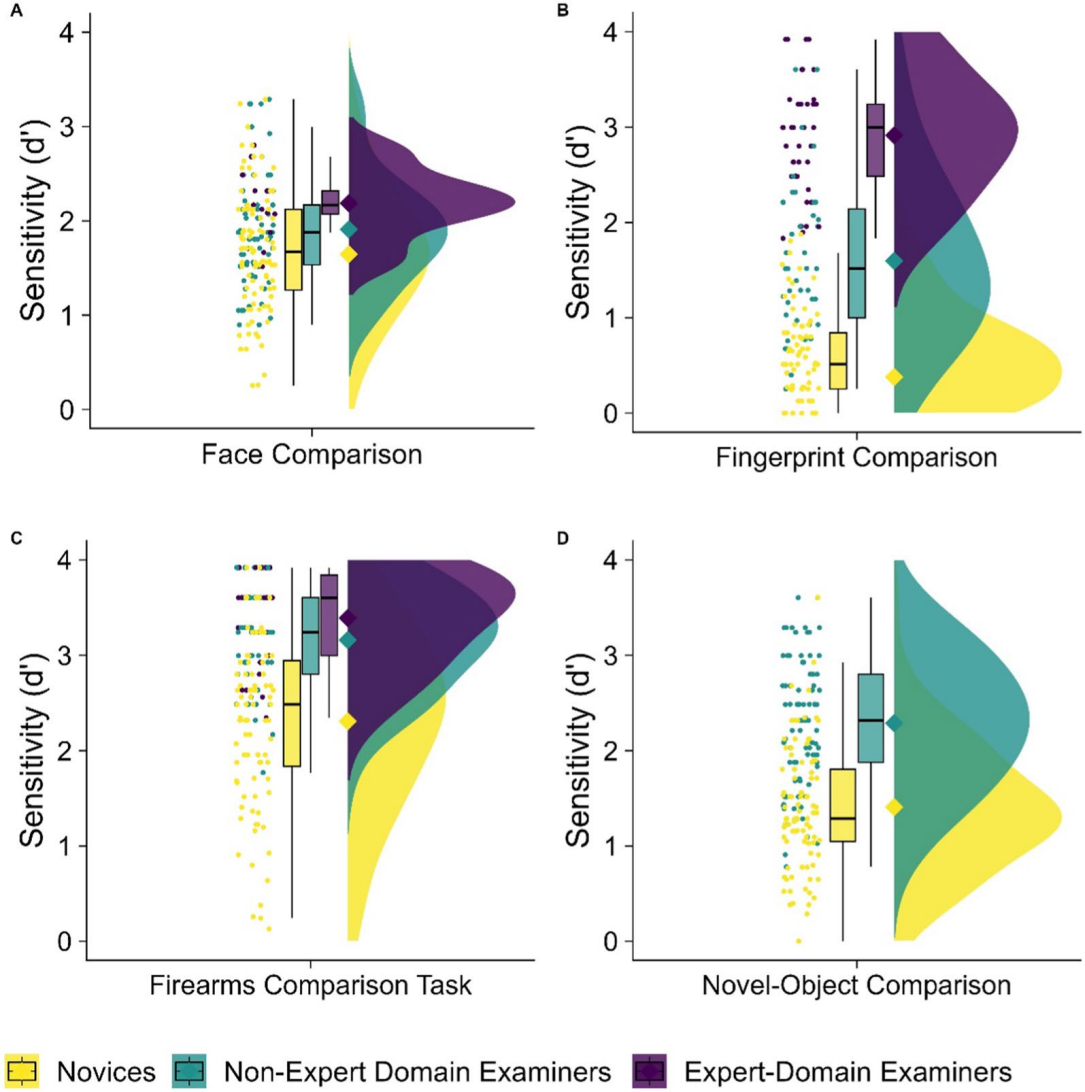
Face comparison performance between the three groups is seen in Panel (**A**), fingerprint comparison performance in Panel (**B**), firearms comparison performance in Panel (**C**), and novel-object comparison in Panel (**D**). Raincloud plots depict (left-to-right) raw jittered data points, box-and-whisker plots, means (represented by diamonds), and frequency distributions. Novices are represented in yellow, non-expert-domain examiners (e.g., face and firearms examiners in fingerprint comparison) in teal, and expert-domain examiners (e.g., fingerprint examiners in fingerprint comparison) in purple. Examiners show superiority across visual comparison tasks outside their domain of expertise, but the most advantage is always found in tasks with examiners’ domain of expertise

*Face comparison*: Face comparison sensitivity differed significantly between the three groups (see Panel A in Fig. 3; *F*_(2, 175)_ = 6.63, *p* = .002). Face examiners (*M* = 2.19, SD = .37) outperformed novices (*M* = 1.65, SD = .67) in face comparison (*t*_(25.28)_ = 4.39, *p* < .001, 95% CI [.29, .79], *d* = .84), as well as fingerprint and firearms examiners (*M* = 1.91, SD = .57; *t*_(23.81)_ = 2.28, *p* = .032, 95% CI [.03, .53], *d* = .51). Fingerprint and firearms examiners also significantly outperformed novices (*t*_(161.89)_ = 2.73, *p* = .007, 95% CI [.07, .45], *d* = .42). These results suggest that all examiners outperformed novices in face comparison, but face examiners outperformed fingerprint and firearms examiners.

*Fingerprint comparison*: Fingerprint comparison sensitivity significantly differed between the three groups (see Panel B in Fig. 3; *F*_(2, 175)_ = 215.60, *p* < .001). Fingerprint examiners (*M* = 2.91, SD = .61) outperformed novices (*M* = .38, SD = .62) in fingerprint comparison (*t*_(80.50)_ = 22.36, *p* < .001, 95% CI [2.31, 2.76], .79], *d* = 4.13), as well as face and firearms examiners (*M* = 1.60, SD = .81; *t*_(77.65)_ = 8.47, *p* < .001, 95% CI [1.01, 1.63], *d* = 1.83). Face and firearms examiners also outperformed novices in fingerprint comparison (*t*_(65.25)_ = 8.74, *p* < .001, 95% CI [.94, 1.50], *d* = 1.78). These results suggest that all examiners outperformed novices in fingerprint comparison, but fingerprint examiners outperformed face and firearms examiners.

*Firearms comparison*: Firearms comparison sensitivity significantly differed between the three groups (see Panel C in Fig. 3; *F*_(2, 175)_ = 33.88, *p* < .001). Firearms examiners (*M* = 3.39, SD = .48) outperformed novices (*M* = 2.31, SD = .94) in firearms comparison (*t*_(97.52)_ = 8.28, *p* < .001, 95% CI [.82, 1.34], *d* = 1.27), as well as face and fingerprint examiners (*M* = 3.16, SD = .53; *t*_(65.28)_ = 2.03, *p* = .047, 95% CI [.01, .46], *d* = .54). Face and fingerprint examiners also outperformed novices in firearms comparison (*t*_(145.85)_ = 7.06, *p* < .001, 95% CI [.61, 1.09], *d* = 1.05). These results suggest that all examiners outperformed novices in firearms comparison, but firearms examiners outperformed face and fingerprint examiners.

*Novel-object comparison:* Novel-object comparison sensitivity significantly differed between groups, as all examiners combined (*M* = 2.29, SD = .67) outperformed novices (*M* = 1.41, SD = .64; *t*_(173.04)_ = 8.93, *p* < .001, 95% CI [.69, 1.07], *d* = 1.34). These results suggest that examiners in all groups outperform novices in an entirely unfamiliar comparison task.

#### Discriminant validity tasks

*Intrinsic motivation:* Intrinsic motivation significantly differed between novices and all forensic examiners (*M* = 4.80, SD = .89) had significantly lower intrinsic motivation than novices (*M* = 5.15, SD = .89; *t*_(174.67)_ = 2.64, *p* = .009, 95% CI [.09, .62], *d* = .40). These results suggest that examiners were less intrinsically motivated than novices during the study and suggest that examiners’ visual comparison advantage cannot be attributed to higher intrinsic motivation.

*Statistical learning:* Statistical learning did not significantly differ between novices (*M* = .48, SD = .19) and forensic examiners (*M* = .53, SD = .22; *t*_(167.49)_ = 1.46, *p* = .147, 95% CI [− .11, .02], *d* = .22). This suggests that the examiners’ advantage in visual comparison does not extend to statistical learning, providing evidence of divergent validity.

### Exploratory individual difference analyses

**Fig. 4:**
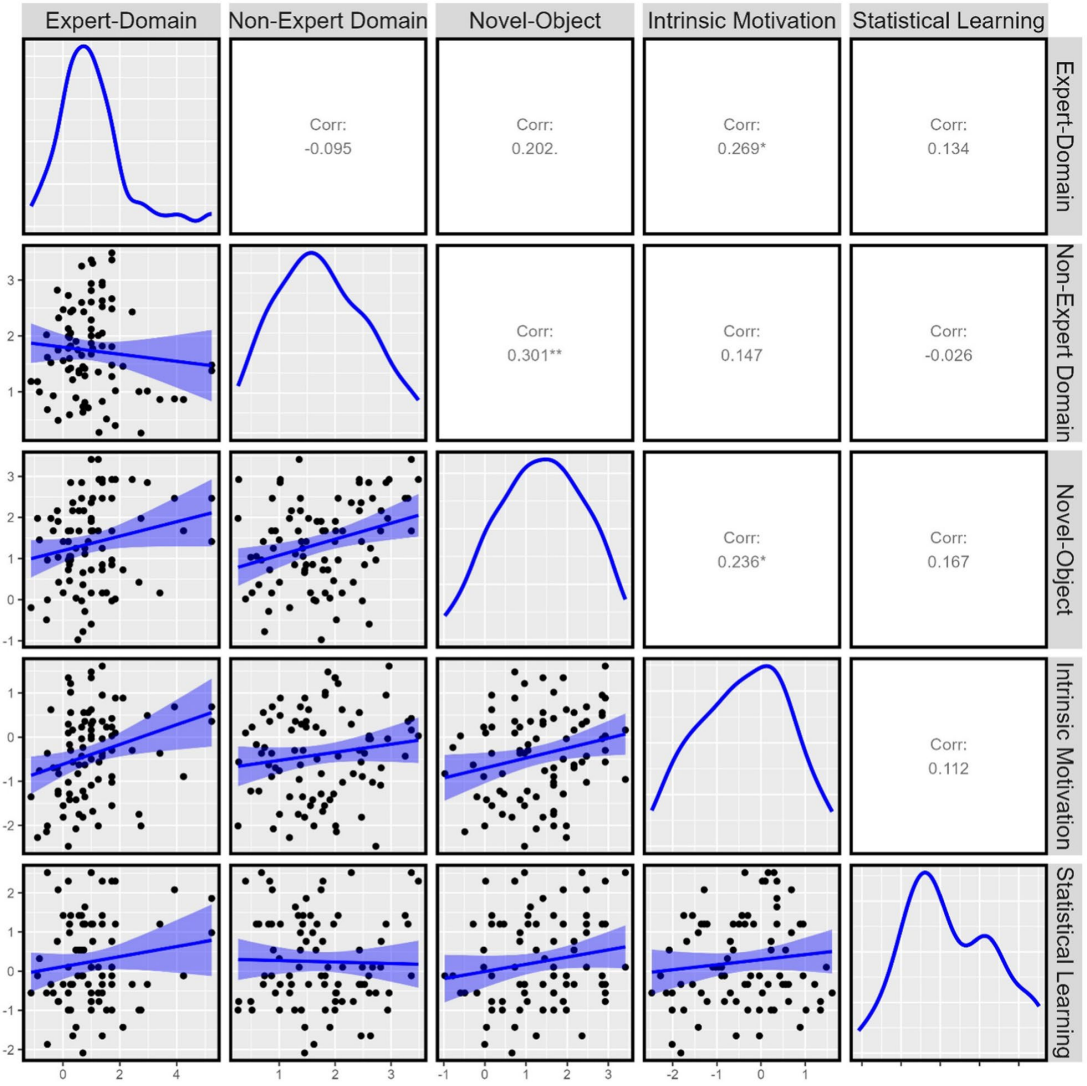
Pearson correlations for examiners for sensitivity on between expert-domain, non-expert-domain, novel-object comparison, and performance on both discriminant validity measures (intrinsic motivation and statistical learning) tasks. Displayed with 95% confidence bands, *p < .05; **p < .001

Examiners’ visual comparison sensitivity outside their domain (i.e., non-expert-domain examiners) significantly correlated with their sensitivity in the novel comparison task (*r* = .301, *p* = .005; see Fig. 4). The observed Bayes Factor of 9.78 provided substantial evidence in favour of the observed correlation (Wetzels et al., 2011). Examiners’ sensitivity within and outside their domain did not significantly correlate with one another (*r* =  − .09, *p* = .389, BF_10_ = 0.35) and the observed Bayes Factor provided anecdotal evidence for the absence of a correlation. Sensitivity within examiners’ domain also did not significantly correlate with novel-object sensitivity (*r* = .202, *p* = .064, BF_10_ = 1.25) and the observed Bayes Factor provided anecdotal support for the presence of a correlation. Within-domain sensitivity was significantly correlated with intrinsic motivation (*r* = .269, *p* = .013, *BF*_10_ = 4.57), but no other correlations were significant.

## Discussion

We explored the relationship between domain-general visual comparison and expertise by comparing the expert-domain and non-expert-domain performance of forensic examiners from three disciplines (faces, fingerprints, and firearms). Examiners in all three disciplines had a distinct domain-specific advantage: within their own domain, examiners outperformed both novices and examiners outside their domain. That is, fingerprint examiners outperformed everyone in fingerprint comparison, firearms examiners outperformed everyone in firearms comparison, and face examiners outperformed everyone in face comparison.

Forensic examiners’ perceptual expertise also generalised. Outside their own domain, examiners outperformed novices in visual comparison, both in forensic tasks outside their area of speciality and entirely novel stimuli. For example, fingerprint examiners outperformed novices in face and firearms comparison, despite these tasks being outside their area of expertise. This generalisation of perceptual expertise is consistent with other examples of generalisable expertise: trained musicians’ skill generalises to speech segmentation (François et al., 2014) and skilled athletes’ abilities generalise to other sports (e.g., baseball to cricket, see Moore & Müller, 2014; and hockey to soccer, see Smeeton et al., 2004).

Yet, our results also revealed a paradox. We identified substantial support for a relationship between individual differences in visual comparison amongst experts outside of their domain (i.e., non-domain and novel-object comparison). This replicates the relationship seen in visual comparison where ability generalises across different tasks (Growns et al., 2022a, 2023; Phillips et al., 2018). While examiners were better than novices outside of their domain of expertise at a group level, examiners' expert domain performance was a poor predictor of individual differences in their ability outside their domain of expertise.

We identified only anecdotal support for relationships between domain-specific skill and performance on both non-domain and novel-object comparison. So how can their expertise generalise if they do not have any shared variance? One potential explanation for this is that acquired expertise reduced the variability of expert-domain performance, reducing the power of its predictive validity by restricting the range of scores. Experience and training may disrupt the relationship between individual differences in these tasks amongst experts (Curby & Gauthier, 2010; Wong et al., 2009).

The source of forensic examiners’ generalisable skill remains unclear. It is possible that forensic examiners self-select into professions they already possess innate aptitude (Growns et al., 2022a, 2023). Alternatively, visual comparison tasks may share core similarities that allow examiners to transfer strategies they learn in one discipline to tasks in other disciplines. Forensic examiners may develop specialised information processing strategies that facilitate both their superior performance and their skill generalisation. Supporting this, fingerprint examiners’ eye-gaze patterns are more consistent with one another than novices’ (Busey et al., 2011). If visual comparison tasks do share core similarities, examiners may harness specialised information processing techniques to excel in tasks outside their domain (see also Dunn et al., 2022). Future research could utilise eye-tracking methodology to investigate how forensic examiners sample information during domain-specific and domain-general visual comparison (Brams et al., 2019).

These results also add to growing evidence of a domain-general visual comparison ability (Growns et al., 2022a; Phillips et al., 2018). Yet, the psychological mechanisms underpinning visual comparison are only beginning to emerge. Several cognitive processes have been implicated in forensic expertise, including domain-specific statistical learning (Busey et al., 2016; Growns & Martire, 2020a; Growns et al., 2022b; Martire et al., 2018), domain-specific visual search (Robson et al., 2021; Searston & Tangen, 2017a; Thielgen et al., 2021), featural processing (Thompson & Tangen, 2014; Towler et al., 2017; White et al., 2015b), and memory retention (Busey & Vanderkolk, 2005; Thompson & Tangen, 2014; for review see Growns & Martire, 2020b; Growns & Neal, 2022). Yet, the relative contribution of these cognitive processes in both the development of expertise and generalisable visual comparison skill is not yet known. Future research should investigate the shared cognitive and perceptual mechanisms underpinning this skill.

Together, these results highlight both the domain-specific and domain-general nature of forensic feature-comparison expertise. Consistent with contemporary models of expertise (Ericsson, 2007; Ericsson et al., 1993; Keith & Ericsson, 2007), forensic examiners displayed perceptual expertise within their own domain of training and experience. Examiners may learn key domain-relevant information that contributes to this superior domain-specific skill—for example, diagnostic information key to specific stimuli (Growns & Martire, 2020a; Growns et al., 2022b, 2022c; Martire et al., 2018; Towler et al., 2021).

These results also have important applied implications. Forensic examiners possess a key capability that could generalise to performance advantages outside their key domain of expertise. However, our data clearly show that domain-specific skill lends the greatest performance boost. Thus, it would be imprudent to recommend that examiners practice outside the discipline that they are trained in—particularly given the high-stakes nature of their judgements within the criminal justice system. Further, our individual difference results suggest that one important way professional performance in forensic science could be improved is by recruiting new forensic trainees based on innate talent. Some forensic organisations have already begun to use screening tests to identify and recruit top-performers in face recognition to work in forensic roles involving face comparison (Dunn et al., 2023; Nador et al., 2022; Robertson et al., 2016; White et al., 2015a).

It is important to note that further research is vital to understanding the generalisation of perceptual expertise in forensic feature comparison. We were not able to recruit our intended sample size of experts in this study—something that is not uncommon in many studies recruiting specialist or expert populations (Martire & Kemp, 2018; Shen et al., 2014). We thus adapted our pre-registered analysis plan, and the data in this study should thus be interpreted with caution. Nevertheless, it is important to note that the number of experts recruited in this study was comparable to other research recruiting forensic examiners (Busey & Vanderkolk, 2005; Growns & Martire, 2020a; Growns et al., 2022b; Martire et al., 2018).

Another important factor to consider is potential differences in motivation between groups and between tasks. While we attempted to control for differences in motivation by rewarding top-performers in all tasks and do not believe this meaningfully impacted our pattern of results as novices were not aware that examiners participated in the study (Ma et al., 2014), it is still possible that examiners were more motivated within their expert-domain tasks than others. Although research has only investigated the impact of motivation on visual comparison performance in novices (Moore & Johnston, 2013), future research should examine how motivation is shaped by expertise.

This study offers novel evidence of the domain-specific and domain-general nature of the perceptual expertise of forensic feature-comparison examiners. Face, fingerprint, and firearms examiners outperform all others within their domain of expertise, but all examiners outperform novices in tasks outside their usual discipline. These results have both theoretical implications about the domain-general nature of perceptual expertise, as well as important applied implications for decision making in forensic science.

## Appendix

See Table 3.

**Table 3:** Psychometric properties of each task (Cronbach’s alpha, skewness, and kurtosis)

	Cronbach’s *α*	Skewness	Kurtosis
Face comparison	.55	− .08	3.49
Fingerprint comparison	.85	.44	2.21
Firearms comparison	.81	− .96	3.84
Novel-object comparison	.68	.16	2.39
Statistical learning	.82	.28	2.45
Intrinsic motivation	.92	− .27	2.44

Note Cronbach’s alpha was calculated on raw accuracy scores per participant for all tasks

## Footnotes

1 118 participants were initially recruited through the snowball-sampling method but per our pre-registered exclusion criteria, only practitioners who reported specialising in face, fingerprint, or firearms examination were retained for analysis to ensure the homogeneity within the sub-groups of the professional sample (*n* = 33 participants were thus excluded using this criteria).

2 Note these trial types are also often referred to as ‘mated’ and ‘non-mated’ pairs. See Biedermann (2022) for discussion.
